# Supplementary material for: Are carbon emissions trading and green financial instruments synergistic? -Comprehensive quantitative research based on content analysis
Source: PLoS One. 2024 Mar 7;19(3):e0298601. doi: 10.1371/journal.pone.0298601 (PMC10919601; doi:10.1371/journal.pone.0298601)
Supplement: S1 Data — (ZIP) [file pone.0298601.s001.zip › Data/地级市Z_F工作报告.zip!/╜¡╬≈/╛░╡┬╒≥╩╨/└┤╫╘╣1⁄2╓┌║┼í╛┬φ┐╦╩2╛▌═°í┐_2012.docx]

各位代表：

　　现在，我代表市人民政府向大会作工作报告，请予审议，并请市政协委员和列席会议的同志提出意见。

　　过去五年，我们的工作取得了很大的成绩，主要表现是：

　　经济总量不断做大。2010年，全市生产总值达到461.5亿元，比2005年增长1倍；财政总收入53.1亿元，增长2.6倍；固定资产投资439.6亿元，增长4倍；规模以上工业增加值160亿元，增长2.3倍，陶瓷总产值160亿元，增长5.5倍；农业总产值57.6亿元，增长88%；旅游总收入66.6亿元，增长2.2倍；开放型经济和工业园区建设快速推进。

　　重点项目取得突破。光伏、陶瓷、汽车零部件和家电等一批产业项目落户。新电厂提前建成；天然气工程全面完工；新的输变电工程投入营运，基本上再造了一个景德镇电网；机场新航站楼投入使用；浯溪口水利枢纽工程和九景衢铁路获得国家批准立项。

　　城市建设步伐加快。城市框架逐步拉开，城区面积由72平方公里扩大到90平方公里；白鹭大桥、民窑遗址博物馆、景东大道等城市建设重点项目相继完工；城市生态环境不断改善，连续四年被评为全省城市环境定量考评第一名，荣获了国家园林城市、中国优秀旅游城市、全国绿化模范城市等荣誉称号。

　　社会事业协调发展。民生工程扎实推进，城乡人均收入分别达到16657元和6521元；社会保障水平大幅提高，城市低保标准保持全省第二，农村低保水平逐年提升；保障性住房建设成效明显，累计建房面积超过70万平方米；社会保持和谐稳定，治安好转率连续三年全省第一；科教文卫体等各项事业都取得了新成就。

　　各位代表，这些成绩是省委、省政府和市委领导下全市人民辛勤劳动的结果。在此，我代表市人民政府感谢市人大的依法监督和市政协的积极参政议政，并向广大干部群众，向各位人大代表、政协委员，向驻景部队指战员、武警、消防官兵、公安民警和社会各界人士表示衷心的感谢！

　　需要指出的是，当前我们的经济总量仍然偏小，发展方式还比较粗放，面临着既要科学发展又要转变方式的双重任务；社会管理和文化建设面临许多新的难题；城市规划、建设和管理水平较低；法治政府建设尚需进一步加强。

　　各位代表，今后五年政府工作的总体要求是：坚持发展第一，人民至上，继承创新，聚精会神地建设繁荣和谐魅力瓷都。在具体工作中，我们将着力处理好一脉相承和与时俱进的关系。一脉相承就是政府工作将保持连续性，一届接着一届干；要坚持发展，用发展解决发展中的问题；坚持以经济建设为中心，特别要努力提高现代工业化水平；坚持以城市和新农村建设为依托，不断改善民生，提升人民尊严和幸福感。与时俱进就是政府工作要体现时代性和创新性；进一步解放思想，转变不适应新时期、新阶段的理念和思路；改进落后的方法和措施；改革不符合实际的机制和体制。

　　今后五年政府工作的主要目标是：经济总量、规模以上工业增加值、财政收入、居民收入实现翻番；生态环境质量保持全省领先，万元GDP能耗下降16%，主要污染物排放总量持续下降；基本公共服务达到全省先进水平。为此，我们将重点做好以下工作。

　　一、抓好经济发展

　　（一）提升工业发展水平和竞争力

　　发展工业，招商引资是龙头，培育新兴工业和提升传统工业是重点内容，园区是平台，基础设施是前提条件，日常调度和运营是不能忽视的工作。

　　1、把招商引资工作摆在发展工业的龙头位置来抓。工业发展靠项目，项目需要投入，投入必须引资。今后五年，我们开展招商引资的出发点是获取效益，着力点是引进项目，基本要求是：（1）更加注重招商引资的投入产出，避免单纯利用优惠政策招商，要靠真诚提高招商引资的竞争力，靠良好的服务提高招商引资的竞争力，靠我们的努力工作提高招商引资的竞争力。（2）更加注重招商引资的针对性，要按产业发展实际和需要，有的放矢地招商，避免盲目投资和重复建设。（3）更加注重招商引资的质量和规模并举，在质量与规模发生冲突时，宁可放弃规模也要追求质量，避免重规模、轻质量的现象。（4）更加注重招商引资的对象，要将土地等稀缺公共资源更多地用于引进带动力强的大企业和央企，避免企业规模结构与资源匹配的失衡。（5）更加注重招商引资的组织领导，要建立统一领导、合理分工、行动有序的招商工作格局，避免招商力量分散和项目引进随意。同时，积极实施“走出去”战略，开拓新市场，推动对外贸易稳步发展。

　　2、把提升传统工业和培育新兴工业作为工业发展的重点内容来抓。

　　以陶瓷产业为重点改造提升传统工业。陶瓷是景德镇的命脉。推动陶瓷产业发展是政府义不容辞的责任。我们一定要尽最大努力尽快实现陶瓷业在转型中振兴。未来五年，我们发展陶瓷工业的主要任务是：（1）编制陶瓷业发展中长期规划；（2）努力推进陶瓷管理体制改革；（3）发展高技术陶瓷和高端日用瓷；（4）加强古陶瓷研究和利用，保护和传承非物质文化遗产；（5）壮大陶瓷文化创意产业，打造文化创意龙头企业；（6）加强陶瓷原料基地建设，掌握优质瓷土资源；（7）实施陶瓷品牌发展战略，加强陶瓷知识产权保护，加强陶瓷技术标准的研究、制订与管理；（8）妥善解决艺术陶瓷税收征管问题；（9）完善陶瓷企业治理结构，推动企业上市；（10）建立陶瓷鉴定评估机构；（11）建立陶瓷交易中心和网络营销体系；（12）培养和引进陶瓷专业人才。同时，加快发展汽车、家用电器、煤化工等支柱产业，推进企业技改和创新，激发传统产业活力。

　　以航空产业为重点推动新兴工业发展。未来五年，我们要着力打造景德镇中国直升机研发生产基地，建设直升机总装园、航空零部件园、国际合作园以及直升机研发园。我们将在财税、土地、资金和人才引进等方面给予航空产业大力支持，努力为航空产业发展提供一流的服务；我们要按照政府主导、市场化运作原则，组建民用直升机投资股份公司；我们要大力引进国内外产业关联度高、技术水平先进的知名航空配套企业，加快形成直升机设计研发、总装、航空零部件配套、维修保养、交流合作能力，到2015年，力争航空产业年产值超过700亿元。同时，大力发展光伏、生物和新医药等新兴产业，加快形成产业集群，努力打造千亿产业。

　　3、把园区建设作为构建工业发展的重要平台来抓。园区是我市经济发展的引擎，要率先发展，为全市经济翻番作出贡献。壮大园区经济实力。抓住高新区升格为国家级高新技术产业开发区的有利机遇，做大、做强、做优高新区，到2015年，力争工业总产值达到600亿元，利税50亿元，实现在国家级高新区中争先进位；努力把陶瓷工业园区打造成全国陶瓷产业基地，把乐平工业园区建成全省生物医药特色园区。提高园区土地产出效率。要节约用地，集约用地，合理配置土地资源，留足发展空间，有步骤、有秩序地开发建设园区。坚持园区企业准入制度。对不符合国家产业政策、国家明令淘汰、禁止建设的项目一律不批；对环境污染严重、污染物不能达标排放的项目一律不批；对高耗能、高污染、资源性项目严格执行环境准入条件。

　　4、把基础设施建设作为创造工业发展良好条件来抓。未来五年，我们将开工建设九景衢铁路、皖赣铁路新建双线及电气化改造、六安至景德镇铁路项目；开工建设浯溪口水利枢纽工程，完成城市防洪工程，中心城市防洪标准由20年一遇提高到50年一遇；建设景德镇港综合码头，实施昌江、乐安河航道整治工程；建设祁浮高速公路，完善国防交通战备公路，积极推进绕城高速公路复工，加快国省道和部分县乡道的升级改造，力争80%县道达到三级标准；加快引进航空公司飞机驻场，再新增3条以上国内航线；做好能源保障供应，完善电力基础设施，加快天然气管网建设，实现城市及工业园区天然气全覆盖。

　　5、把加强工业调度和运行作为工业发展的重要工作来抓。加强工业运行监测。我们将完善规模以上工业企业经济运行监测体系，定期发布运行分析报告，为企业提供最新的资源、市场、技术信息，使企业在发展中抢占先机。加强要素保障。加强电力需求管理，加大煤、油、运协调，保障土地、资金、人力。做好跟进服务。实施重大工业项目绿色通道并联审批制度，提高审批效率；更加重视项目跟进服务；支持昌飞、昌汽、焦化、华意等重点企业实施重大项目，加快发展。

　　（二）扎实做好“三农”工作

　　积极发展现代农业。严格保护耕地，粮食播种面积稳定在138万亩，确保粮食生产安全；加大农业综合开发力度，加快高标准农田建设；大力发展有机茶、无公害蔬菜、特色养殖等高效农业；推进农业产业化经营，培育产业化龙头企业和各类农民专业合作社，到2015年，力争市级以上龙头企业达到100家，农民专业合作社达到500家。加强水利基础设施建设。2012年前完成国家规划治理的34座病险水库除险加固任务，2015年前完成347座小二型水库除险加固任务，完成5000亩以上重点堤防整治，维修改造城乡排涝设施。努力增加农民收入。全面落实种粮补贴和农机购置补贴等强农惠农政策；妥善安排好农村富余劳动力，鼓励和引导农民自主创办各种经济实体。加快新农村建设。完善农村基础设施和公共服务体系，不断改善农村生产生活条件。到2015年，力争三分之二以上自然村完成新农村建设目标。加强森林资源培育和保护。强化森林资源管护力度，提高森林火灾及林业有害生物防控综合能力，到2015年确保森林覆盖率达到66%。

　　（三）大力发展现代服务业

　　加快旅游产业发展。我们将依托陶瓷历史文化资源和生态优势，全面提高旅游产业水平。古窑和瑶里要创建国家5A级旅游景区；古县衙、洪岩仙境等要完善景区功能；要发展老城区旅游、古村风貌旅游和陶瓷文化体验游；加强“六山两湖”区域合作，扩大客源，打造景德镇-婺源-三清山等精品旅游线路；规划建设游客集散和服务中心；鼓励研发具有地方特色的旅游商品。支持金融服务业发展。要利用基金、股票和债券等金融工具增加企业资金来源；加快发展集合信托、短期融资券等，拓宽中小企业直接融资渠道；发展城乡保险业务；推动知名会计师、律师事务所和投资咨询公司等中介机构落户景德镇。促进物流服务业发展。科学布局和建设市县两级现代物流中心，培育现代化物流龙头企业。推动会展商贸服务业发展。办好每年的国际陶瓷博览会，提升会展经济水平；进一步完善商贸业发展专项规划，加快建设中心城区特色商业街区，发展生活服务业，吸引人气，培育商气，聚集财气。

　　（四）统筹发展县域经济

　　我们将综合运用财税和土地等政策，支持县（市、区）发挥特色，扬优成势。乐平市重点发展生物医药、矿产、建材、无公害蔬菜、食品；浮梁县重点发展旅游、生态农业、绿色食品和高科技陶瓷；昌江区要壮大城郊经济，配套新兴产业，发展现代服务业；珠山区重点发展商贸物流、文化娱乐等服务业。在发展县域经济的同时，我们将按多元化的要求开展小城镇建设，重点建设一批工业型、旅游型、工矿型、城郊型、商贸及农副产品加工型小城镇。

　　二、抓好城市建设

　　未来五年，景德镇城市建设基本原则是，既体现瓷都传统文化，又展现城市现代风貌，不求最大，但求最好，避免以快为荣。城市建设基本目标是，三年明显进步，五年巨大变化，努力把景德镇建设成为文化之城、历史名镇和生态家园。

　　（一）科学规划城市

　　城市之美源于规划。城市先进是规划的先进，城市只有规划好才能建好管好。完善规划体系。城市总体规划要凸显城市定位，明确发展目标，充分发挥其对各项建设的宏观指导和调控作用；同时，做好城市分区规划、控制性详规、专业规划和城市设计，处理好局部与整体、近期与长远、城建与环保、开发建设与历史保护等关系，努力实现城市规划的系统性和可操作性。维护规划权威。规划制定应广泛听取社会各方面的意见，特别要听取专家的意见；规划应有法定约束力，一经批准，必须严格执行，违者问责！要建立完善城市规划管理的监督机制，加强城市规划的执法，提高规划的监管水平。

　　（二）着力建设城市

　　未来五年，城市建设的重点内容是，美化一条江，打造新老两个城区，开发三条道路，建设四个中心。

　　一是开发建设好新城区。要把江上旅游、江岸美化和岸边棚户区改造结合起来，美化一条昌江；要通过定制招标和成片开发，建设好昌南、景德、景东三条道路两侧区域；要建设“四个中心”：（1）建设行政中心，盘活公共资产，辐射带动新城区发展；（2）建设陶瓷创意中心，为集聚国际国内陶瓷创意人才搭建平台；（3）建设商务会展中心，为国际瓷博会和陶瓷交易以及购物休闲提供现代化场所；（4）建设文化传媒中心，为广播影视、歌舞戏曲等文化事业发展提供基地。用5至10年时间，把新城区建设成为规划有序、形态优美、功能完善、产业聚集、社会和谐的现代新区。

　　二是保护利用好老城区。老城保护利用基本要求是，保护历史文脉，改善群众生活，发展文化旅游，完善城市功能。老城保护利用重点内容是，（1）加快老城区古、旧风貌建筑的保护利用，疏散过度密集人口；（2）建设御窑国家考古遗址公园，保护利用其周边街区和民居；（3）保护利用三闾庙等三个古码头，恢复古商业风貌；（4）保存传统生活方式和民风习俗，留住城市历史记忆；（5）保存现代工业遗存。

　　另外，在城市建成区要加快基础设施建设，提升水电气保障能力；特别要舍得把钱花在城市地下，重视城市地下管网的规划、建设和管理，重点实施城市排污、地下给排水管网建设工程，解决建成区内涝之患。

　　（三）严格管理城市

　　未来五年城市管理主要工作是，（1）打击“两违”。要加大整治集体土地私买私卖和乱搭乱建的违法行为，区、乡、村一级抓一级，严格实行问责制度，对工作不力的责任人要依法依规从严处理，下大决心和大气力坚决铲除违法用地和违章建设顽疾。（2）治理“七乱”。要加大对乱停、乱倒、乱吐、乱行、乱挂、乱摆和乱涂等陋习的综合治理力度，规范城市秩序，树立城市形象。（3）创新管理。扎实做好城市创建工作；落实城市管理行政综合执法责任制；进一步明确城市区划及其管理机构；解决城市管理部门职能交叉、多龙治水、管理缺位问题；利用数字化城市管理等现代信息技术，不断提高城市管理水平。

　　三、抓好社会管理

　　（一）转变社会风气

　　当前我们的社会风气中存在着不讲文明礼貌、不遵守公共秩序、不讲社会公德等现象，这些都对城市形象和声誉造成了极大的负面影响。改善社会风气是当前社会管理的迫切任务。我们要激发群众的责任感，像爱护自己的眼睛一样爱护自己的幸福家园，努力营造“瓷都是我家，共建惠大家”的社会风气。要提高市民道德素养。全面推进社会主义核心价值体系建设，深入开展社会公德、家庭美德、个人品德教育，不断把道德规范转化为人民群众的内在品质和自觉行动；加大新闻媒体对不文明现象曝光力度，引导市民讲文明，讲礼貌。要强化市民法制观念。教育和引导人们依法表达利益诉求，不能因为谋取个人利益而损害公众利益和公共安全，克服信闹不信法等不良习惯；下大力气治理非法聚众拦门堵路问题；坚决打击恶意扰乱社会秩序的违法行为。

　　（二）维护社会稳定

　　健全维稳工作机制。完善利益诉求保障和矛盾调处机制，充分发挥人民来访接待中心的作用，畅通民意表达渠道，切实做好重复访化解工作；健全公共突发事件应急处置机制，加快推进武警支队现代化建设，提高应急处理能力；实行重大决策社会稳定风险评估，防止群体性事件的发生；完善社会治安防控体系，切实增强群众安全感。加强互联网管理。掌握网络动态，防止网络炒作，疏导社会情绪。加强社区管理。社区要更多地参与社会管理，不断提高化解纠纷、维护稳定的能力，做到小事不出社区，大事不出街道，矛盾不上交；社区服务要以民生需求为导向；社区文化建设要以增强凝聚力为宗旨。加强安全保障。树立安全发展理念，落实责任，推进重点行业领域安全生产专项整治和隐患排查治理，加强学校、公众聚集场所、道路交通、食品药品等安全监管，防止重特大事故发生。

　　（三）发展社会事业

　　把教育放在基础性、先导性、战略性地位。全面推进素质教育，促进九年义务教育均衡发展，加快普及高中阶段教育，大力发展职业教育和学前教育，关心特殊教育和老年教育；努力实现国家要求的教育财政性支出占GDP比重的目标。推进科技进步。加快推进国家创新型城市试点工作，大力实施科技创新“六个一”工程，开展航空制造、陶瓷新材料、生物医药等重点领域技术攻关，培育一批创新型企业和科技领军人才。完善公共卫生服务体系。建设基本医疗保障制度，实施国家基本药物制度，健全基层医疗卫生服务体系，促进基本公共卫生服务逐步均等化，推进公立医院改革试点。发展文化体育等各项事业。丰富人民群众文化生活；发展群众性体育运动，提高竞技运动水平，增强人民群众身体素质；抓好人口和计划生育工作，统筹解决人口问题，促进人口均衡发展；加快推进国防后备力量和民兵预备役部队建设。抓好民生工程。积极解决高校毕业生、农村转移劳动力、城镇就业困难人员就业问题；推进覆盖城乡居民的社会保障体系建设；加强房地产市场调控，加快保障性住房建设，改善居民居住条件；发展社会福利和慈善事业；努力稳定市场物价，落实“菜篮子”市长负责制；加快农村“渡改桥”工程建设，推进农村客运建设，坚定不移地优先发展城市公共交通。

　　四、抓好法治政府建设

　　（一）政府要坚持依法行政。要实行公众参与、专家论证、集体讨论的重大决策机制，做到会前广泛酝酿，会上深入讨论，会后严格执行；要建立健全行政制度体系，完善行政执法程序，严格规范公正文明执法；要健全行政权力运行监督机制，做到权力事项清、权力流程清、权力责任清，有权必有责、用权受监督；扎实推进网上审批和电子监察系统建设；加强审计监督，强化领导干部经济责任审计、工程审计和专项资金审计；加强行政管理绩效考核，落实政府问责制度，建立依法行政的激励机制；要严守“七条”财经纪律：（1）依法加强财税管理，严格执行国家税收政策，不准违规操作；（2）依法加强财政大额专项资金管理，完善资金绩效评估机制，提高资金使用效益；（3）依法加强政府性债务和政府融资平台公司管理，完善政府债务偿还机制，积极防范债务风险；（4）依法加强民生工程和节能减排等专项资金管理，坚决防止截留挪用专项资金；（5）依法加强行政机关经费管理，厉行节约，防止铺张浪费；（6）依法规范统计调查行为，加强统计数据管理，严肃统计纪律，确保统计数据的合法性、真实性；（7）依法加强土地管理，不准非法批地、未批先用、批少用多，依法依规管好地、用好地、造好地。

　　（二）公务员要做到勤、实、廉。我们的公务员作风上仍然存在一些问题，突出表现就是“庸懒散虚”。庸就是能力平平，业务不精。懒就是追求安逸，不肯动脑，不肯动手。散就是自由散漫，有令不行，有禁不止。虚就是说假话虚话，办假事虚事。我们将以治理庸懒散虚为重点，抓好队伍建设，努力使公务员做到：

　　一要勤。我们将围绕提升工作效率搞出几条硬措施，教育、批评和处理不谋事、不干事，好事揽自家、坏事赖人家、难事推上家、做事找下家的人，使各级公务员以只争朝夕、时不我待的危机意识和一日无为、三日不安的责任意识，勤学习、勤思考、勤工作。

　　二要实。讲真话是对信仰的忠诚。说假话的人走俏得利会让真诚做人、老实干事者感到寒心。我们一定要旗帜鲜明地提倡讲真话，反对讲假话。我们一定教育各级公务员讲实话，出实招，办实事，求实效，不做轰轰烈烈的表面文章，不提脱离实际的高标准，不喊哗众取宠的空口号，不搞劳民伤财的假政绩。

　　三要廉。我们的行政领导机关及其公务员要遵守政治、人事和财经纪律，绝不能腐化堕落、贪污受贿；权力集中部门、资金密集行业、民生领域要改善行风，绝不能搞吃、拿、卡、要，更不能违法乱纪；行政执法机关绝不能以案谋私、贪赃枉法和为黑恶势力充当“保护伞”；相关行政机关及其公务员在重大责任事故和群体性事件中绝不能失职渎职。公共资源属于人民，我们一定要倍加珍惜，做到不以公谋私，而要廉洁奉公。

　　公务员队伍中存在的问题，人民群众看得最清，感受最深。我们一定要认真倾听人民群众的呼声，从人民群众反映最强烈的问题抓起，从人民群众最不满意的地方改起，从人民群众最盼望的事情做起，积极回应社会和群众的关切。我们愿意接受全市人民的监督！

　　各位代表，历届班子的不懈努力，为景德镇的发展积累了宝贵的精神财富，打下了扎实的物质基础。我们已看到一轮喷薄欲出的朝阳，我们已眺望到遥远海中的希望桅杆。在中国特色社会主义的伟大旗帜下，有省委、省政府和市委的坚强领导，我们有决心，用科学发展这把钥匙，去开启千年古镇的振兴之门！我们有信心，用悠久厚重的陶瓷文化和重振雄风的丰硕成果把“景德镇”这三个享誉世界的大字擦得更加璀璨夺目！让我们用意志、情怀和热血谱写一曲建设繁荣和谐魅力瓷都的壮丽篇章！
